# Development and Validation of a Cuproptosis‐Based Risk Score Model for Predicting Neoadjuvant Chemotherapy Response in Breast Cancer: A Transcriptomic Analysis

**DOI:** 10.1155/tbj/6097777

**Published:** 2026-05-29

**Authors:** Lihai Zhang, Jiao Wang, Baihong Tan, Xingquan Wang, Zhenglong Luan

**Affiliations:** ^1^ Department of General Surgery, The First Affiliated Hospital of Jiamusi University, Jiamusi, 154003, Heilongjiang, China, jmsuf1.cj68.com; ^2^ Department of Pediatric, The First Affiliated Hospital of Jiamusi University, Jiamusi, 154003, Heilongjiang, China, jmsuf1.cj68.com; ^3^ Graduate School, Jiamusi University, Jiamusi, 154007, Heilongjiang, China, jmsu.edu.cn

**Keywords:** breast cancer, cuproptosis, immune microenvironment, neoadjuvant chemotherapy, risk score model

## Abstract

**Objective:**

This study developed a cuproptosis‐related transcriptomic risk score model to predict neoadjuvant chemotherapy (NAC) response in breast cancer (BC) patients and explored its association with the tumor immune microenvironment.

**Methods:**

Analysis of transcriptomic and clinical data from TCGA and GEO revealed differentially expressed cuproptosis‐related genes. LASSO‐based Cox regression was used to build the risk score. Model performance was evaluated using Kaplan–Meier survival, ROC curves, and GSEA/GSVA in the training cohort and further validated in an independent external cohort. Drug sensitivity was predicted using the oncoPredict tool, and RT‐qPCR was used to validate key gene expression.

**Results:**

A 10‐gene prognostic model was developed based on the identification of 71 cuproptosis‐related genes. The risk score correlated with survival outcomes, PAM50 subtypes, tumor stage, and pathologic response. It showed good predictive performance in both training (AUC = 0.719) and testing (AUC = 0.689) cohorts. Key genes (CIRBP, INPP4B, IL6ST, and CCL20) were validated and linked to NAC response.

**Conclusion:**

The cuproptosis‐based risk score model effectively predicts NAC response and may guide personalized treatment in BC. It also reveals the relevance of cuproptosis‐related genes in immune modulation and chemotherapy sensitivity.

## 1. Introduction

Breast cancer (BC) remains one of the most prevalent malignancies among women globally [1], with a high incidence and marked variability in clinical outcomes. Its molecular heterogeneity, marked by different subtypes such as Luminal A, Luminal B, HER2‐positive [2], and triple‐negative BC (TNBC) [3], results in differing therapeutic responses and prognoses. Despite therapeutic advances, accurately predicting individual responses to treatment—particularly to neoadjuvant chemotherapy (NAC), a standard option for locally advanced BC—remains a major clinical challenge. Current biomarkers, though informative, do not fully capture the complexity of treatment response, underscoring the need for novel predictive indicators [4].

Cuproptosis, a copper‐driven cell death, is emerging as a key focus in cancer research [5]. Cuproptosis, unlike apoptosis and ferroptosis, is induced by copper binding to lipoylated components in the tricarboxylic acid cycle, resulting in proteotoxic stress and cell death [6, 7]. Recent reviews have highlighted that cuproptosis represents a mechanistically distinct form of regulated cell death compared to classical pathways, particularly ferroptosis, and is closely associated with mitochondrial metabolism and copper‐dependent cellular toxicity, thereby implicating a potential role in tumor therapeutic vulnerability [8, 9]. Furthermore, emerging evidence indicates that exploiting these distinct metabolic vulnerabilities may enhance tumor sensitivity to conventional chemotherapy and potentially help overcome drug resistance in cancer treatment [10, 11]. Although its role has been investigated in several malignancies, including prostate and liver cancers, its role in BC remains underexplored [12]. Several cuproptosis‐related genes (e.g., LIAS, LIPT1, FDX1, and PDHA1) have been implicated in regulating this process [13, 14], but their association with BC subtypes and potential predictive value for chemotherapy response are not yet clearly defined [15].

Importantly, one of the pressing challenges in NAC administration is the heterogeneity in patient responses [16]. Previous studies have demonstrated that genetic signatures involving cell death pathways, immune regulation, and tumor microenvironment (TME) characteristics can influence chemotherapy efficacy [16, 17]. Immune cell infiltration in the TME plays a central role in treatment outcomes. Nevertheless, whether cuproptosis‐related genes contribute to immune–tumor interactions and can be harnessed to predict NAC efficacy is still uncertain.

We built a cuproptosis‐based transcriptomic risk score model to predict NAC response in BC patients. By integrating multicohort gene expression data from The Cancer Genome Atlas (TCGA) and Gene Expression Omnibus (GEO), we identified differentially expressed cuproptosis‐related genes among BC subtypes, evaluated their association with treatment response (pathologic complete response [pCR] vs. residual disease [RD]), and constructed a prognostic model. We further analyzed how cuproptosis‐related gene expression correlates with immune microenvironment and drug sensitivity, with the goal of improving individualized treatment strategies for BC.

## 2. Materials and Methods

### 2.1. Data Sources

Transcriptomic and clinical data for BC were obtained from the TCGA‐BRCA dataset and GEO datasets (GSE25055, GSE25065, and GSE25066). RNA‐seq data in fragments per kilobase million (FPKM) from TCGA were normalized using log2 (FPKM + 1) transformation. GEO data were processed using the same normalization strategy to ensure interdataset consistency.

### 2.2. Differential Expression Analysis

Limma in R was used for differential expression analysis after normalizing the transcriptomic data. The empirical Bayes method was applied to moderate variance estimates. A linear model was fit to compare gene expression between patients with pCR and those with RD across the entire cohort, with a contrast matrix defined accordingly. Significant differential expression was defined as |log2 fold change (logFC)| > 0.585 and *p* < 0.05, with results displayed in volcano plots and heatmaps.

### 2.3. Cox Proportional Hazards Regression

The prognostic relevance of cuproptosis‐related genes was assessed using univariate Cox regression to determine their link with distant recurrence‐free survival (DRFS). Genes with *p* < 0.05 were included in a multivariate Cox regression to identify independent prognostic factors. Least absolute shrinkage and selection operator (LASSO) regression was applied to select the most predictive genes for the final model. A risk score model was then developed by weighting the expression levels of selected genes with their corresponding Cox regression coefficients.

### 2.4. Functional Enrichment Analysis

To elucidate the biological relevance of survival‐related genes, the clusterProfiler R package was used for Gene Ontology (GO) and Kyoto Encyclopedia of Genes and Genomes (KEGG) enrichment analyses, with statistical significance determined by hypergeometric testing and corrected using the Benjamini–Hochberg method. Terms with a false discovery rate (FDR) < 0.05 were considered significantly enriched.

### 2.5. Risk Prediction Model

Transcriptomic data from the GSE25066 dataset (*n* = 488) with DRFS information were obtained from GEO. After excluding samples with missing or invalid follow‐up data, stratified sampling divided the cohort into a 70% training set and a 30% testing set. Feature selection in the training set was performed using LASSO–Cox regression, with the optimal *λ* selected based on the minimum cross‐validation error using 10‐fold cross‐validation (cv.glmnet function). A multivariate Cox model was applied to estimate regression coefficients for genes with nonzero coefficients. The risk score was calculated as follows: Risk score = ∑(βi × Expri), where βi is the Cox coefficient and Expri is the gene expression.

The maxstat.test method was used to determine the optimal cutoff for stratifying patients into high‐ and low‐risk groups. Kaplan–Meier curves and receiver operating characteristic (ROC) analysis were used to evaluate model performance, with independent validation conducted in the testing set.

### 2.6. Clinical Correlation Analysis

Clinical characteristics from GSE25055 and GSE25065 were integrated with risk scores. Appropriate statistical tests (e.g., Wilcoxon, Kruskal–Wallis, and chi‐squared) were used to assess associations between risk groups and clinical variables such as tumor grade, stage, and molecular subtype (PAM50).

### 2.7. Gene Set Enrichment Analysis (GSEA)

clusterProfiler was used for GSEA with Molecular Signatures Database (MSigDB) reference gene sets, enrichment scores were computed, and 1000 permutations were performed to assess statistical significance.

### 2.8. Gene Set Variation Analysis (GSVA)

GSVA was applied to quantify pathway activity scores using MSigDB gene sets. A nonparametric kernel estimation method (kcdf = “Gaussian”) was used to compute enrichment scores across individual samples. Results were visualized as bar plots representing enriched pathways.

### 2.9. Immune Infiltration Analysis

Immune cell fractions in the TME were estimated using the LM22 matrix in CIBERSORT. Comparisons of immune infiltration were performed between risk groups and progression‐free interval (PFI) status. Correlation analyses were conducted to assess relationships between immune cells and clinical variables.

### 2.10. Nomogram Construction

A nomogram was developed using the rms R package, incorporating the risk score to predict individual patient survival. Accuracy was assessed through calibration curves and time‐dependent ROC, with performance quantified by AUC.

### 2.11. Drug Sensitivity Prediction

OncoPredict R, utilizing the GDSC database, was used to infer drug sensitivity. Estimated IC_50_ values were calculated for various anticancer agents. Correlation analyses between risk score and predicted drug sensitivity were performed to identify potential therapeutic options.

### 2.12. RT‐qPCR Validation

MCF‐7 (ATCC HTB‐22), BT‐474 (HTB‐20), MDA‐MB‐231 (HTB‐26), and MCF10A (ATCC CRL‐10317) cell lines were used for RNA extraction with TRIzol reagent (Invitrogen, 15596018). RNA purity and concentration were evaluated using a NanoDrop spectrophotometer (Thermo Scientific). Using 1 μg of total RNA, cDNA synthesis was performed with HiScript III RT SuperMix for qPCR (+gDNA wiper) (Vazyme, R323‐01). qPCR was conducted on a QuantStudio 5 Real‐Time PCR System (Applied Biosystems) with ChamQ Universal SYBR qPCR Master Mix (Vazyme, Q711‐02). Target gene expression was normalized to GAPDH, and relative levels were calculated using the 2^–ΔΔCt^ method. All reactions were performed in triplicate. Primer sequences are listed in Supporting Table 1.

### 2.13. Statistical Analysis

Data analysis was conducted with R (Version 4.1.1) and SPSS (Version 26.0). Continuous variables were presented as mean ± SD for normally distributed data or interquartile range (IQR) for non‐normally distributed data, and categorical variables were presented as counts and percentages. Group comparisons used the *t*‐test, Mann–Whitney *U* test, chi‐square test, or Fisher’s exact test. *p* < 0.05 was considered significant.

## 3. Results

### 3.1. Analysis of Differentially Expressed Cuproptosis Genes in NAC Response

To investigate NAC‐associated transcriptomic alterations in BC, we utilized the GSE25055 dataset for differential expression analysis, comparing patients with pCR and RD. Genes with |logFC| > 0.585 and *p* < 0.05 were defined as significantly differentially expressed. Figure 1(a) shows 616 differentially expressed genes (DEGs), with 368 upregulated and 248 downregulated. A heatmap of the top 50 upregulated and downregulated genes further illustrated distinct expression patterns associated with NAC response (Figure 1(b)). To explore the role of cuproptosis in treatment efficacy, we intersected these DEGs with a curated set of 1079 cuproptosis‐related genes derived from previously published studies and databases, including GeneCards and MSigDB, as reported by Chen et al. [18]. This comparison identified 71 overlapping genes (Figure 1(c)), which were selected for subsequent prognostic modeling and functional analyses.

FIGURE 1Identification of cuproptosis‐related DEGs associated with NAC response in BC. (a) Volcano plot highlighting upregulated (red) and downregulated (blue) genes between pCR and RD in GSE25055 (|logFC| > 0.585, *p* < 0.05). (b) Heatmap of the top 50 upregulated and downregulated DEGs, clustered according to NAC response status. (c) Venn diagram illustrating the overlap between the 1079 cuproptosis‐related genes and the 616 DEGs, revealing 71 cuproptosis‐related DEGs.

**Figure:**
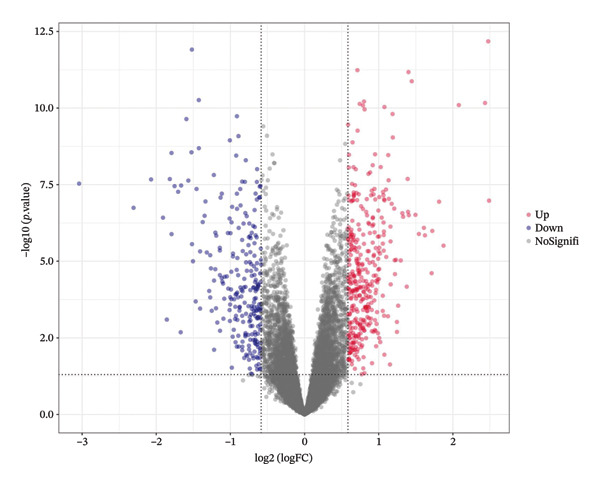
(a)

**Figure:**
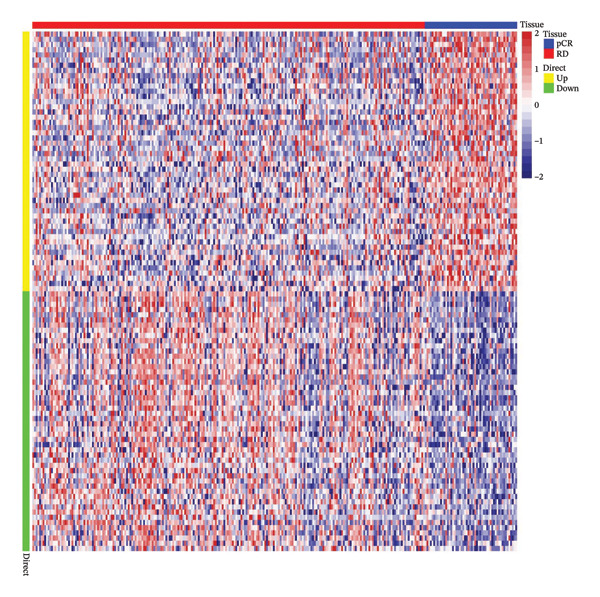
(b)

**Figure:**
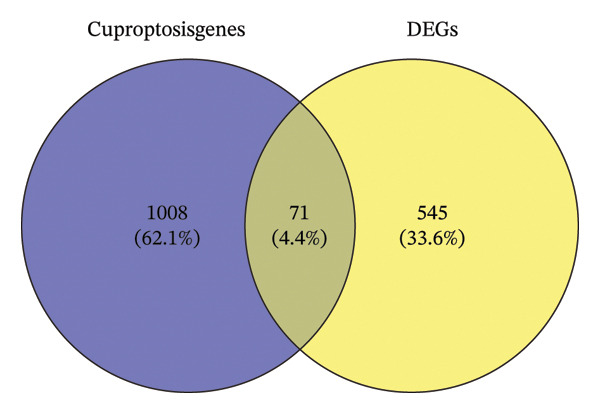
(c)

### 3.2. Univariate Cox Regression for Survival‐Related Genes

To evaluate the prognostic potential of the 71 cuproptosis‐related DEGs, we performed univariate Cox regression analysis based on DRFS. Table 1 presents 41 genes significantly associated with DRFS (*p* < 0.05), which exhibited diverse hazard ratios, included both protective (HR < 1) and risk‐associated (HR > 1) factors, and collectively formed the basis for the construction of the prognostic model.

**TABLE 1:** Univariate Cox regression analysis of 41 cuproptosis‐related genes significantly associated with disease‐free survival in BC patients.

Gene_ID	HR	95% CI (lower)	95% CI (upper)	*p* value
CDK1	1.516	1.151	1.995	0.003
IL6ST	0.629	0.504	0.783	< 0.001
TBX3	0.852	0.760	0.955	0.006
TFF1	0.845	0.772	0.924	< 0.001
GALNT7	0.636	0.511	0.793	< 0.001
RRAGD	1.303	1.073	1.583	0.008
INPP4B	0.692	0.588	0.815	< 0.001
TOX3	0.856	0.779	0.941	0.001
EFHC1	0.811	0.715	0.920	0.001
TNFRSF21	1.328	1.097	1.606	0.004
RACGAP1	1.453	1.096	1.927	0.009
RARRES1	1.198	1.085	1.323	< 0.001
ADM	1.417	1.138	1.765	0.002
UGCG	0.576	0.444	0.747	< 0.001
CCL20	1.286	1.135	1.456	< 0.001
PROM1	1.207	1.064	1.368	0.003
AGR2	0.864	0.809	0.923	< 0.001
DEFB1	1.356	1.157	1.588	< 0.001
CCL2	1.233	1.080	1.407	0.002
CRYAB	1.222	1.051	1.419	0.009
CCL8	1.322	1.122	1.559	0.001
GBP1	1.262	1.080	1.475	0.003
CRIP1	0.824	0.742	0.916	< 0.001
TSPAN13	0.641	0.496	0.827	0.001
TAP1	1.316	1.056	1.641	0.015
VAV3	0.749	0.631	0.889	0.001
CCL4	1.263	1.045	1.527	0.016
PLAT	0.815	0.708	0.939	0.005
HOMER3	1.471	1.114	1.942	0.007
FOLR1	1.301	1.046	1.616	0.018
CA12	0.728	0.618	0.857	< 0.001
ITM2C	1.173	1.018	1.351	0.027
CFD	0.850	0.746	0.970	0.015
CXCL10	1.266	1.097	1.462	0.001
MRPS30	0.671	0.478	0.942	0.021
ABLIM3	0.814	0.702	0.942	0.006
VLDLR	1.563	1.150	2.125	0.004
CRABP1	1.201	1.070	1.348	0.002
KIAA0020	1.572	1.167	2.117	0.003
DNALI1	0.770	0.678	0.875	< 0.001
CIRBP	0.489	0.362	0.659	< 0.001

### 3.3. Enrichment Analysis of Survival‐Related Genes

To investigate the functions of the 41 cuproptosis‐related genes associated with DRFS, GO and KEGG enrichment analyses highlighted notable immune‐related pathways in GO, involving monocyte chemotaxis, chemokine‐mediated signaling, and lymphocyte migration (Supporting Figure 1A). These pathways are critical for immune cell trafficking and activation within the TME, which may directly influence chemotherapy response in BC. KEGG analysis revealed gene enrichment in chemokine signaling, cytokine–cytokine receptor interaction, and related immune signaling pathways (Supporting Figure 1B).

The results suggest cuproptosis‐related genes may modulate immune responses, thereby contributing to differential NAC outcomes.

### 3.4. Construction and Validation of the Cuproptosis‐Based Risk Score Model

Using the 41 cuproptosis‐related genes significantly associated with DRFS, we constructed a prognostic risk model based on the GSE25055 dataset. LASSO Cox regression with 10‐fold cross‐validation was applied to select the most predictive qualities (Figures 2(a) and 2(b)), resulting in the identification of 10 genes with nonzero coefficients (IL6ST, GALNT7, INPP4B, RACGAP1, UGCG, CCL20, DEFB1, CCL8, DNALI1, and CIRBP). These genes were incorporated into a multivariate Cox model to calculate individualized risk scores. Using the optimal cutoff, patients were stratified into high‐ and low‐risk groups. Kaplan–Meier analysis in the training cohort revealed significant DRFS differences (*p* < 0.0001), with time‐dependent ROC curves showing AUCs of 0.78, 0.80, and 0.77 at 1, 2, and 3 years (Figures 2(c) and 2(d)). Model validation in the GSE25065 cohort confirmed its robustness, with significant survival stratification (Figure 2(e)) and AUCs of 0.666, 0.725, and 0.720 at 1, 2, and 3 years (Figure 2(f)).

FIGURE 2Construction and validation of the cuproptosis‐based risk score model. (a) LASSO coefficient profiles of 41 survival‐related genes. (b) 10‐fold cross‐validation for optimal *λ* selection in the LASSO Cox model, identifying 10 feature genes. (c‐d) Kaplan–Meier survival curve and time‐dependent ROC curve for the training set (GSE25055), showing significant survival separation and high predictive accuracy (AUCs = 0.77–0.80). (e‐f) Kaplan–Meier curve and ROC analysis in the testing set (GSE25065), confirming the model’s generalizability.

**Figure:**
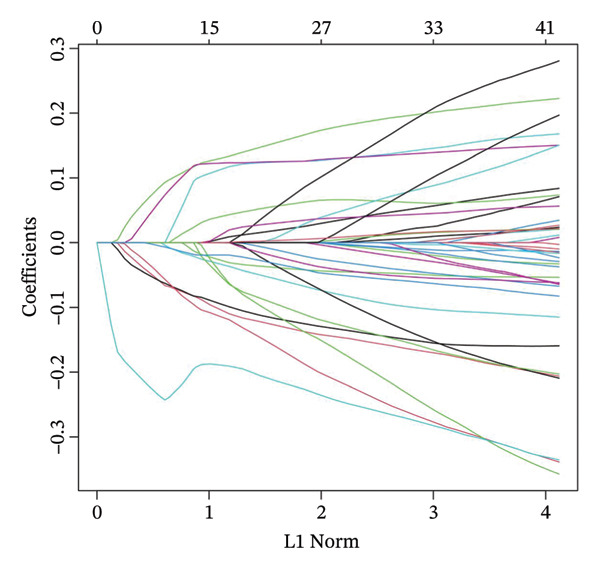
(a)

**Figure:**
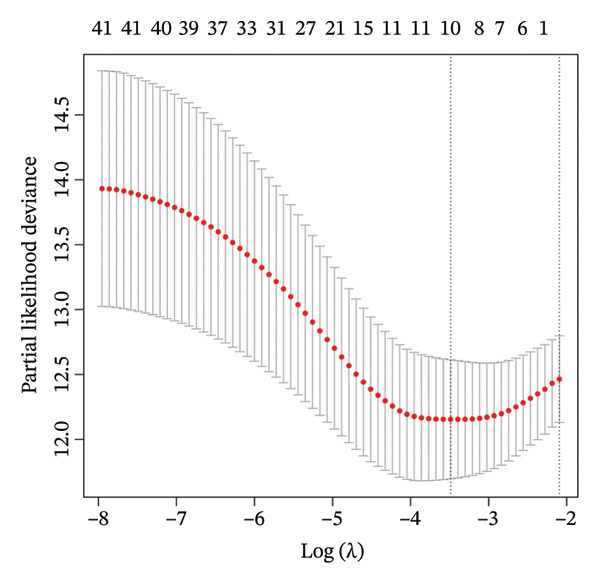
(b)

**Figure:**
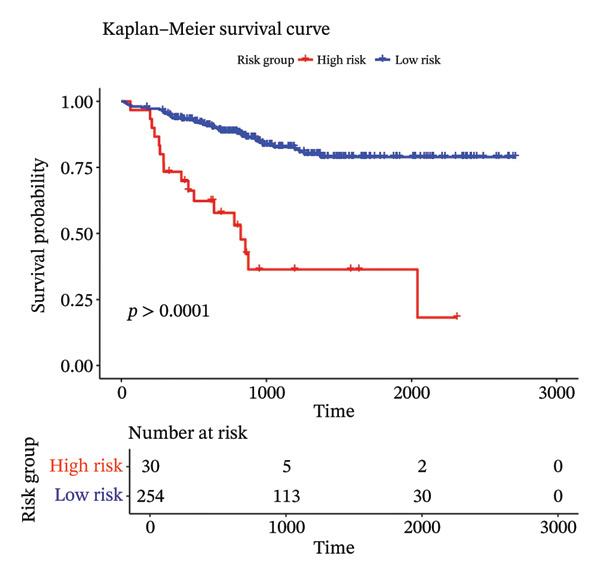
(c)

**Figure:**
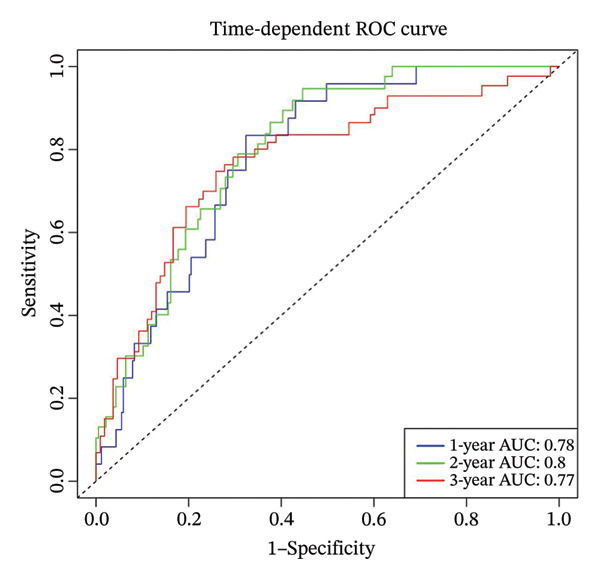
(d)

**Figure:**
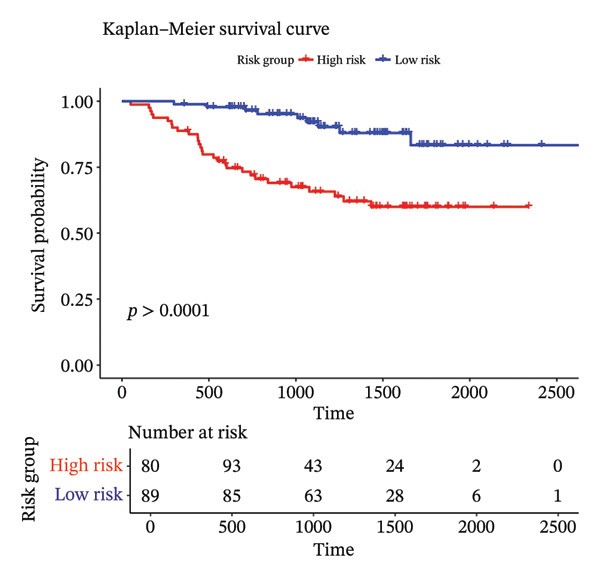
(e)

**Figure:**
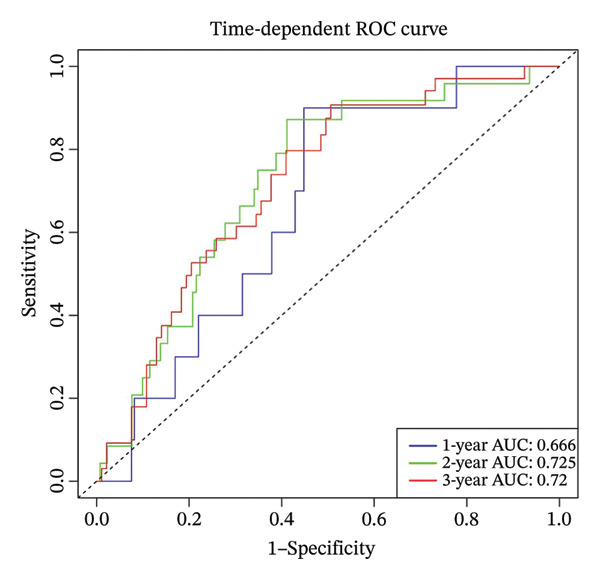
(f)

These results support the model’s potential utility in risk stratification and prognosis prediction for BC patients undergoing NAC.

### 3.5. Biological Pathways Associated With the Risk Score Revealed by GSEA and GSVA

GSEA was applied to risk scores in GSE25055 to explore pathways. GO analysis revealed high‐risk scores were linked to upregulation of immune processes, including regulation of neutrophil activation, while fatty acid oxidation was downregulated (Figure 3(a)). KEGG pathway analysis revealed risk scores were linked to DNA replication and TNF signaling pathways, whereas pathways such as phenylalanine metabolism and tyrosine metabolism were suppressed (Figure 3(b)). To further assess pathway activity variation, GSVA analysis was conducted across risk groups. High‐risk patients had elevated scores in INTERFERON_GAMMA_RESPONSE, INFLAMMATORY_RESPONSE, and other immune activation pathways, while showing reduced activity in P53 signaling and FATTY_ACID_METABOLISM (Figure 3(c)).

FIGURE 3Functional pathway analysis associated with the cuproptosis‐based risk score. (a) GSEA using GO biological processes shows positive enrichment of immune‐related terms (e.g., neutrophil activation) and negative enrichment of metabolic processes (e.g., fatty acid oxidation) in high‐risk patients. (b) GSEA using KEGG pathways identifies enrichment of DNA replication and TNF signaling in the high‐risk group and suppression of amino acid metabolism pathways. (c) GSVA comparing pathway activity between risk groups reveals upregulation of INTERFERON_GAMMA_RESPONSE and INFLAMMATORY_RESPONSE and downregulation of P53 signaling and FATTY_ACID_METABOLISM in the high‐risk subgroup.

**Figure:**
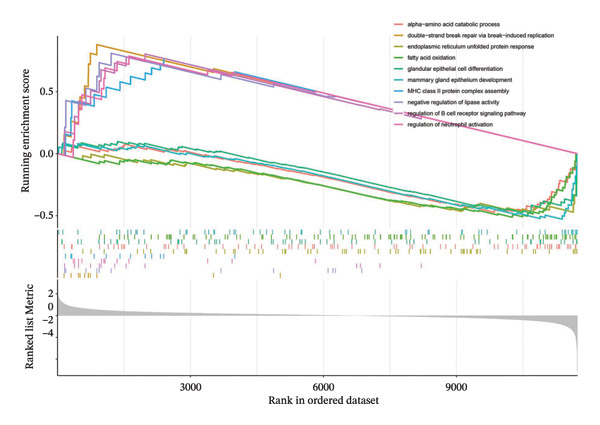
(a)

**Figure:**
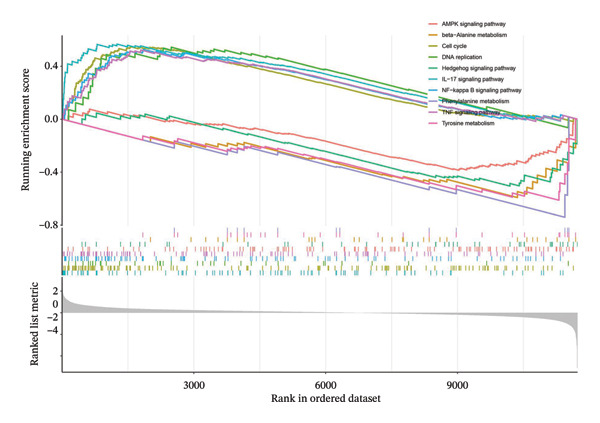
(b)

**Figure:**
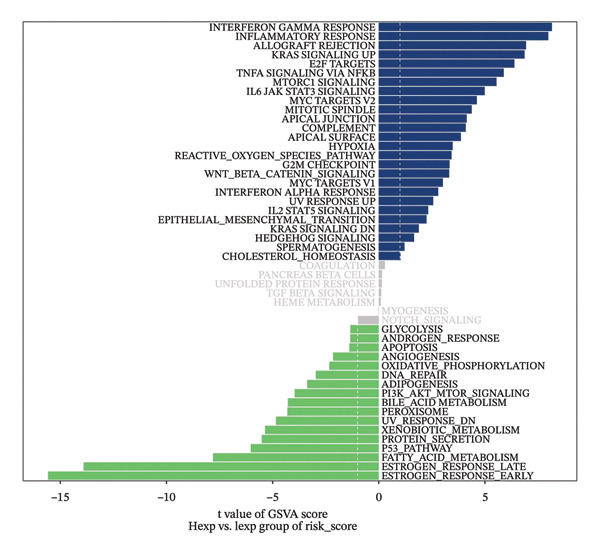
(c)

These findings suggest that cuproptosis‐associated risk scores are closely linked to immune activation and metabolic suppression, reflecting distinct tumor biological behaviors across risk groups.

### 3.6. Immune Cell Infiltration Characteristics Associated With the Cuproptosis‐Based Risk Score

To investigate the relationship between the cuproptosis‐based risk score and the tumor immune microenvironment, we estimated immune cell infiltration using the CIBERSORT algorithm. A total of 22 immune cell subtypes were quantified in each sample of the training cohort. Figure 4(a) shows the immune cell distribution across risk groups. Figure 4(b) illustrates the correlations between immune cell subtypes, highlighting coordinated patterns such as positive associations between T cells and macrophages. Notably, dendritic cells (DCs)—both activated and resting—exhibited significant infiltration differences between risk groups (Figure 4(c)). Correlation analysis revealed significant associations between the risk score and DCs, macrophages, CD4^+^ T cells, and CD8^+^ T cells (Figure 4(d)).

FIGURE 4Immune infiltration landscape and its association with the risk score. (a) Bar plot of 22 immune cell subtypes across high‐ and low‐risk groups based on CIBERSORT analysis. (b) Heatmap illustrating correlations among immune cell subtypes. (c) Boxplot comparing immune cell infiltration levels between risk groups; significant differences were observed in dendritic cells and other subsets. (d) Correlation between risk score and immune cell abundance, highlighting significant associations (^∗^ *p* < 0.05 and ^∗∗^ *p* < 0.01).

**Figure:**
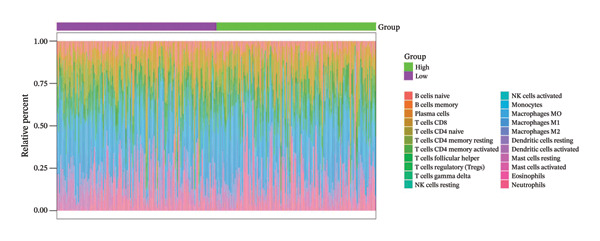
(a)

**Figure:**
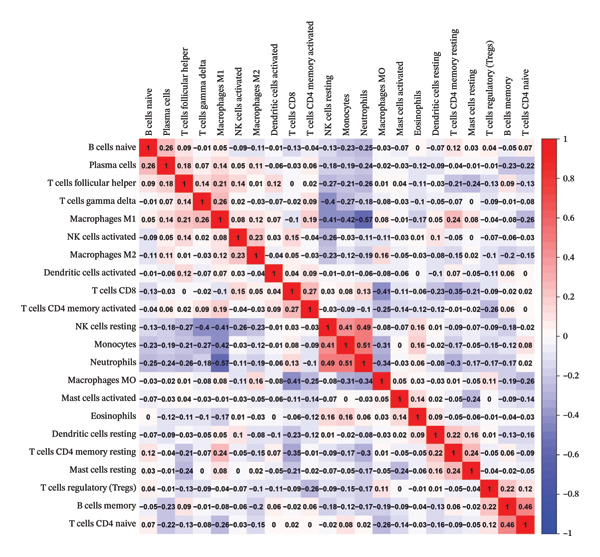
(b)

**Figure:**
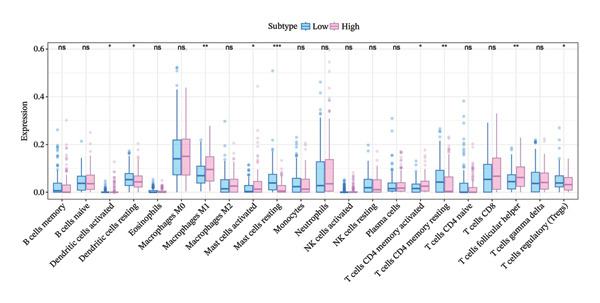
(c)

**Figure:**
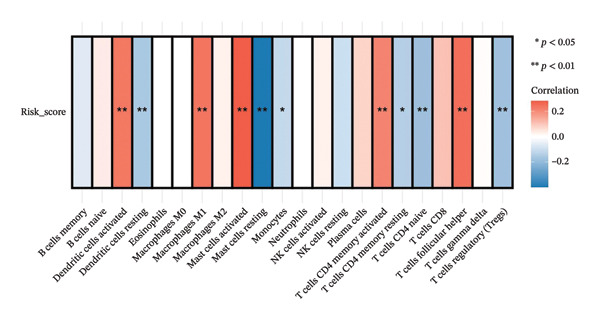
(d)

The prognostic model may reflect the immunological state of the TME, potentially influencing chemotherapy response and survival outcomes.

### 3.7. Clinical Relevance Analysis

The correlation between risk scores and multiple clinical characteristics in the training set (GSE25055) and testing set (GSE25065).

The risk score in the GSE25055 cohort was not significantly associated to age or clinical stage (*p* = 0.839 and 0.100, respectively; Figures 5(a) and 5(c)), but showed significant correlations with tumor grade, PAM50 molecular subtype, pathologic response (pCR), and DRFS (Figures 5(b), 5(f), 5(g), and 5(h)). Associations with T and N stage demonstrated borderline significance (*p* = 0.054 and 0.065, respectively; Figures 5(d) and 5(e)).

FIGURE 5Association between risk score and clinical features in the training set (GSE25055). (a–h) Boxplots showing the distribution of risk scores across age groups, tumor grades, clinical stage, T and N classification, PAM50 subtypes, pathologic response status (RD vs. pCR), and DRFS outcomes. Significant correlations were observed for Grade (*p* = 5.277 × 10^−8^), PAM50 (*p* = 1.095 × 10^−23^), pCR (*p* = 1.866 × 10^−3^), and DRFS (*p* = 4.889 × 10^−4^).

**Figure:**
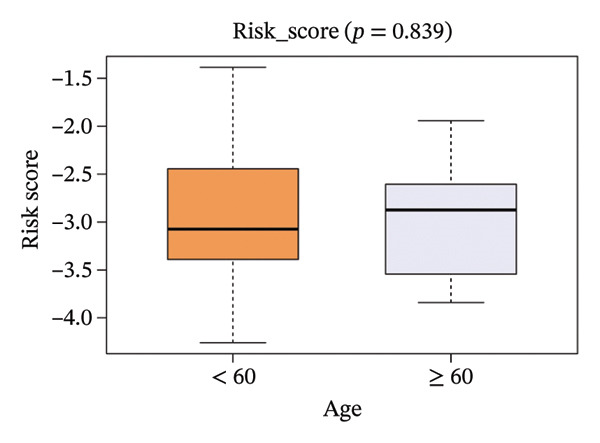
(a)

**Figure:**
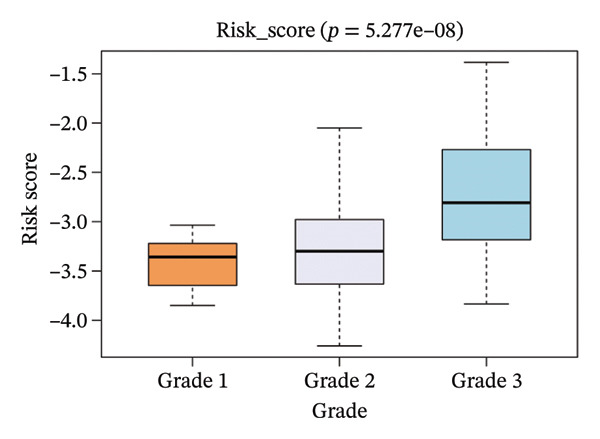
(b)

**Figure:**
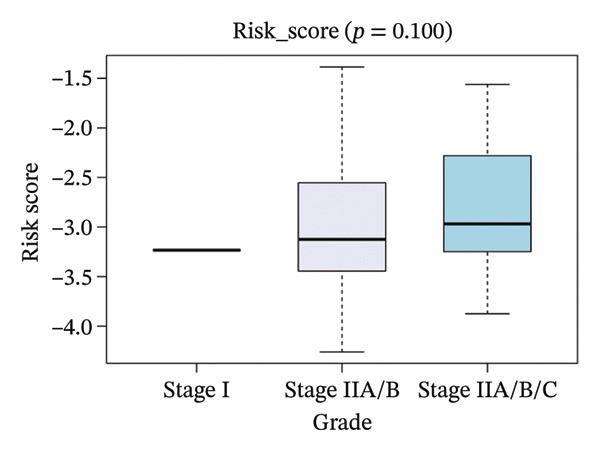
(c)

**Figure:**
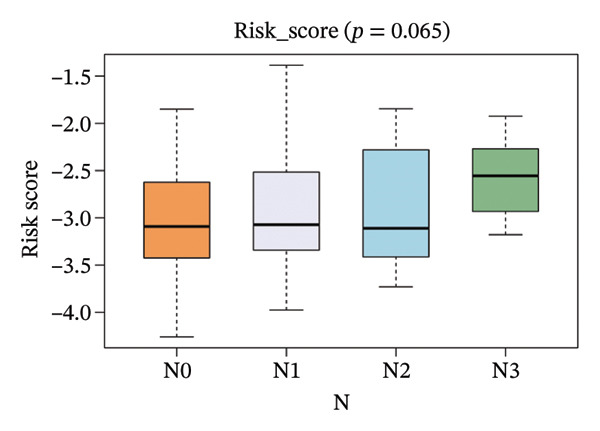
(d)

**Figure:**
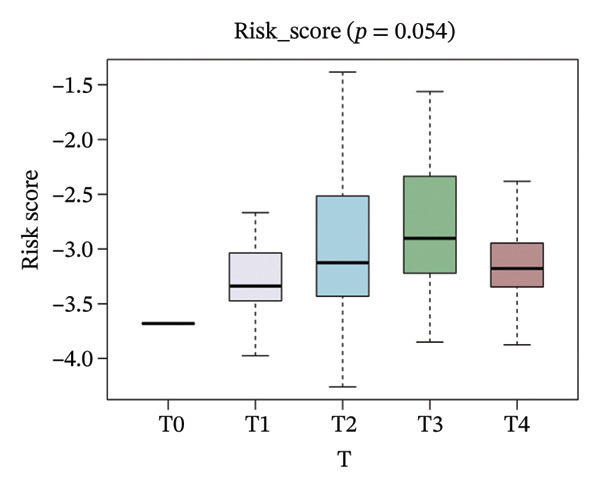
(e)

**Figure:**
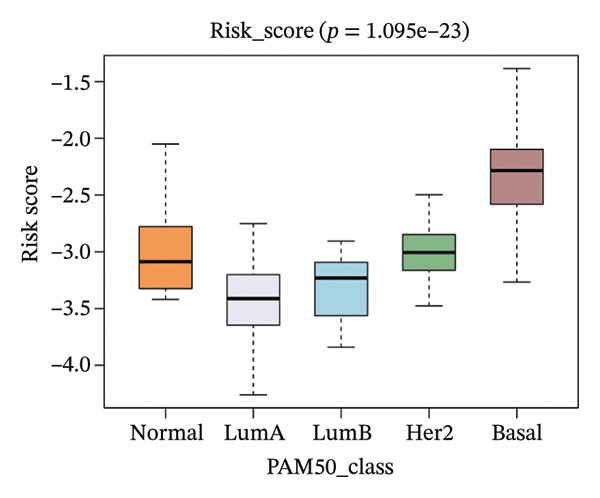
(f)

**Figure:**
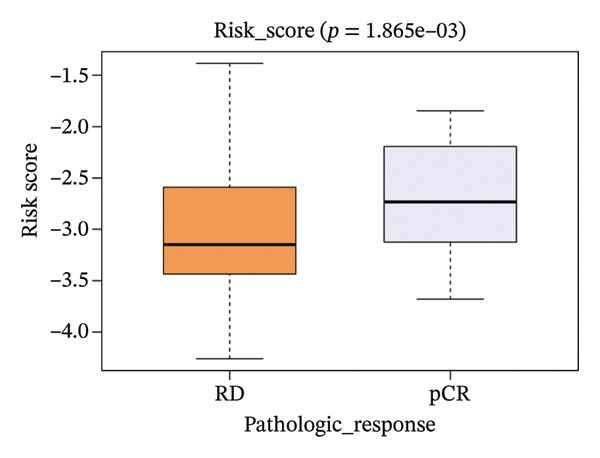
(g)

**Figure:**
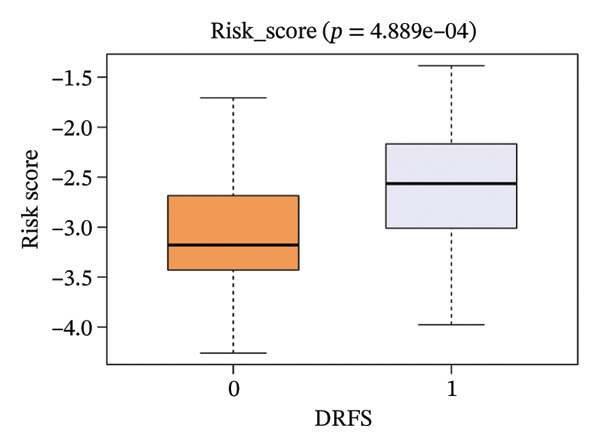
(h)

In the GSE25065 cohort, the risk score remained significantly associated with tumor grade, PAM50 molecular subtype, pathologic response, and DRFS, and was also associated with clinical stage as well as T and N classification (Figure 6). These findings support the risk score as a robust and generalizable predictor of treatment response and prognosis, closely linked to key clinicopathological characteristics across independent datasets.

FIGURE 6Associations of risk scores with clinical variables in GSE25065 (a–h) Boxplots illustrating risk score variations across clinical subgroups, including age (*p* = 0.818), tumor grade (*p* = 1.814 × 10^−21^), clinical stage (*p* = 1.231 × 10^−3^), N stage (*p* = 2.99 × 10^−3^), T stage (*p* = 1.454 × 10^−2^), PAM50 subtype (*p* = 2.438 × 10^−41^), pathologic response (pCR vs RD; *p* = 7.534 × 10^−10^), and DRFS status (*p* = 5.777 × 10^−7^).

**Figure:**
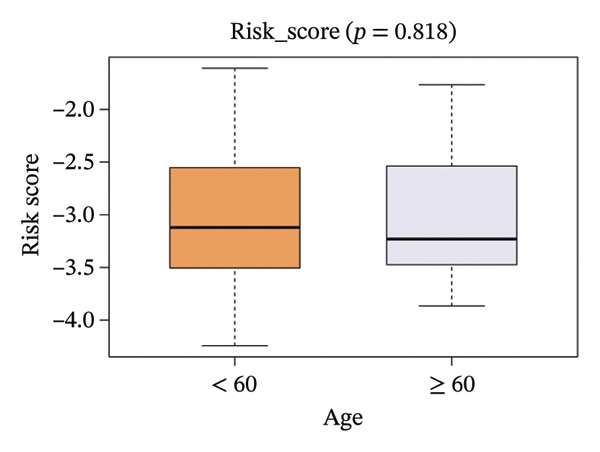
(a)

**Figure:**
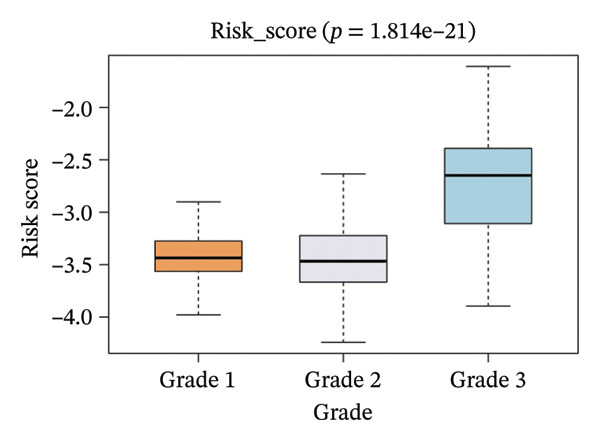
(b)

**Figure:**
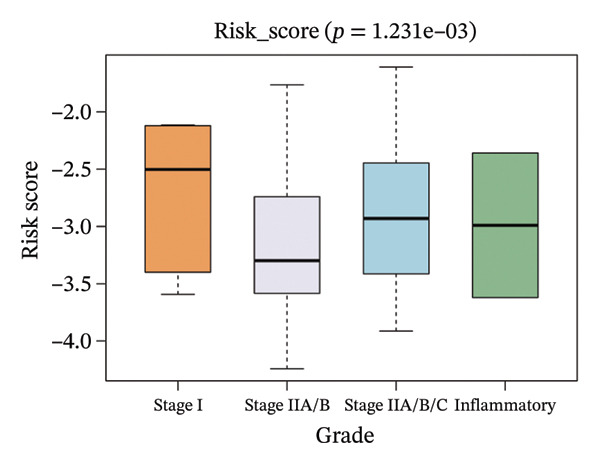
(c)

**Figure:**
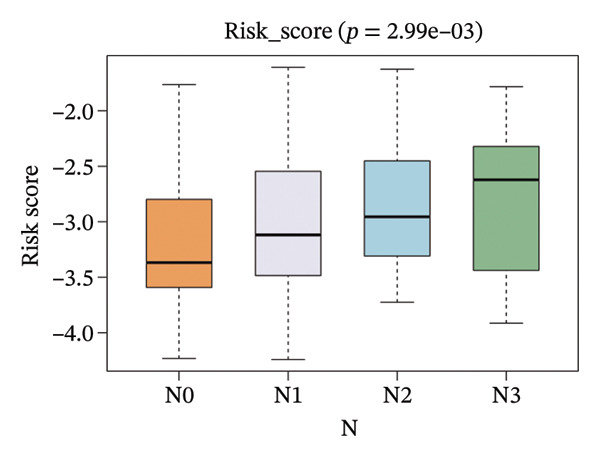
(d)

**Figure:**
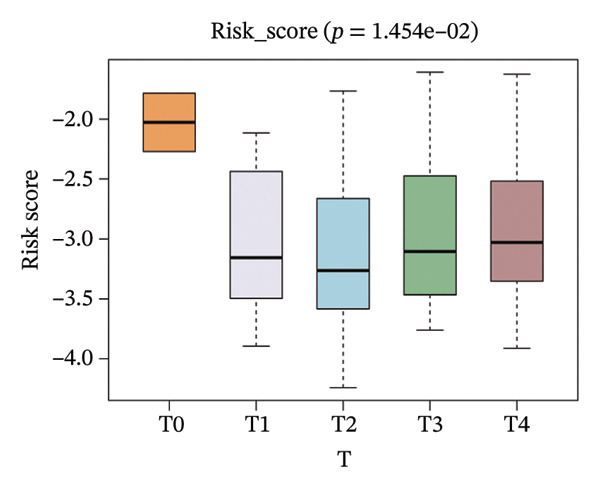
(e)

**Figure:**
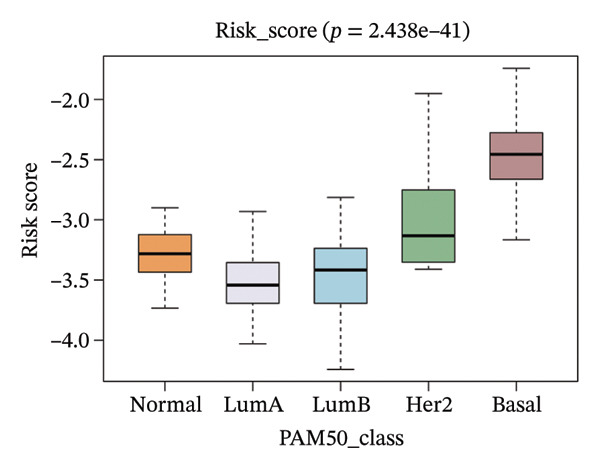
(f)

**Figure:**
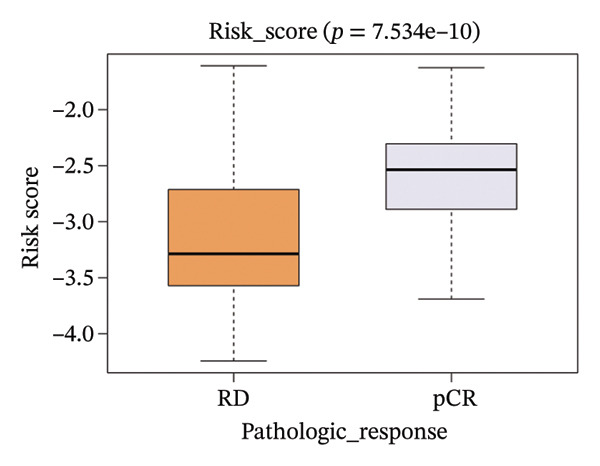
(g)

**Figure:**
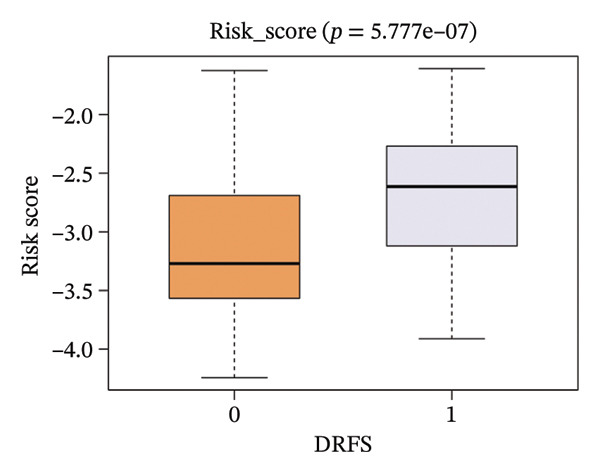
(h)

### 3.8. Personalized Survival Prediction via Nomogram Based on Cuproptosis Risk Score

To facilitate individualized survival prediction, a nomogram based on the cuproptosis‐related risk score was developed using the rms package for both the GSE25055 training cohort and the GSE25065 validation cohort (Figures 7(a) and 7(d)). The nomogram translates each patient’s risk score into 1‐, 2‐, and 3‐year survival probabilities. Model performance was assessed via calibration curves, which revealed strong accord in predicted and observed outcomes (Figures 7(b) and 7(e)), and ROC analysis, which showed moderate discrimination with AUCs of 0.719 in the training set and 0.689 in the validation set (Figures 7(c) and 7(f)).

FIGURE 7Nomogram construction and evaluation for individualized survival prediction. (a, d) Nomograms built using the risk score model in the training (GSE25055) and validation (GSE25065) cohorts, estimating 1‐, 2‐, and 3‐year survival probabilities. (b, e) Calibration curves reveal good alignment in predicted and observed survival. (c, f) ROC curves demonstrating the model’s discrimination ability; AUCs were 0.719 for training and 0.689 for validation.

**Figure:**
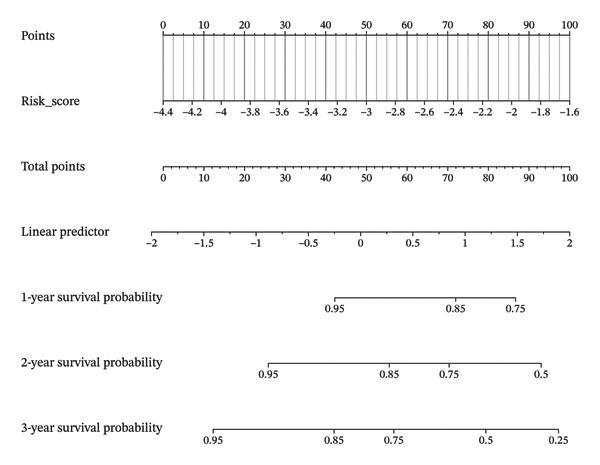
(a)

**Figure:**
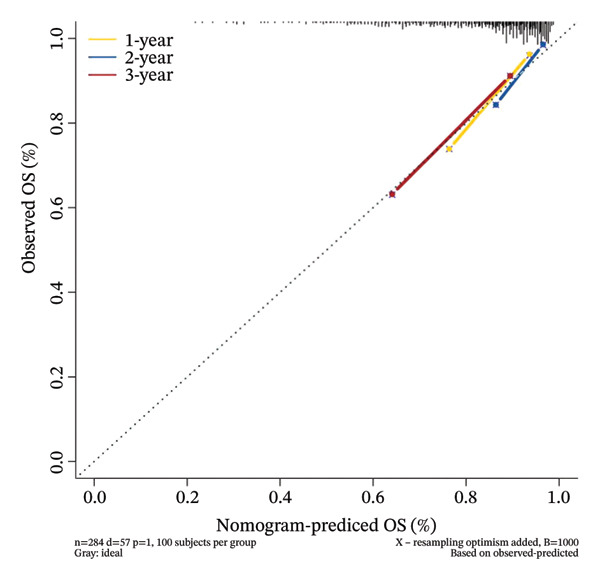
(b)

**Figure:**
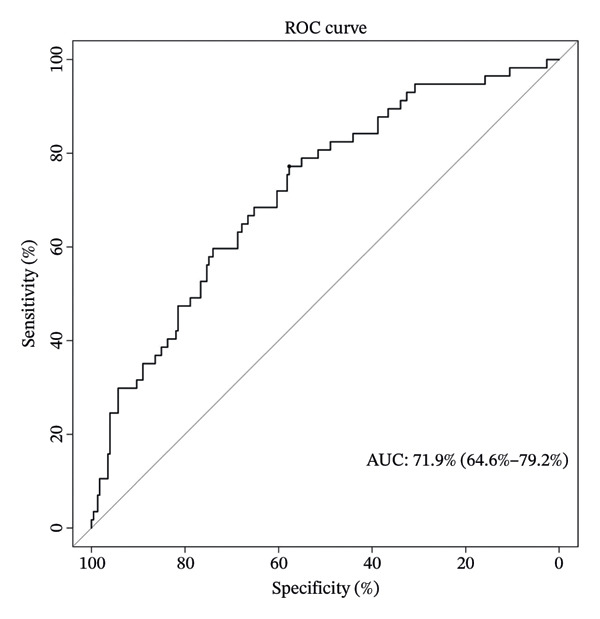
(c)

**Figure:**
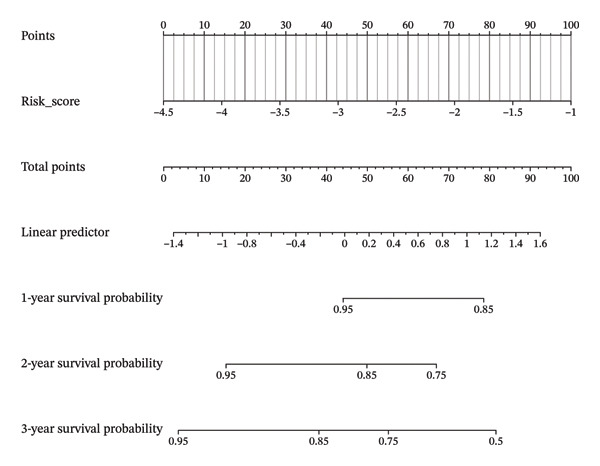
(d)

**Figure:**
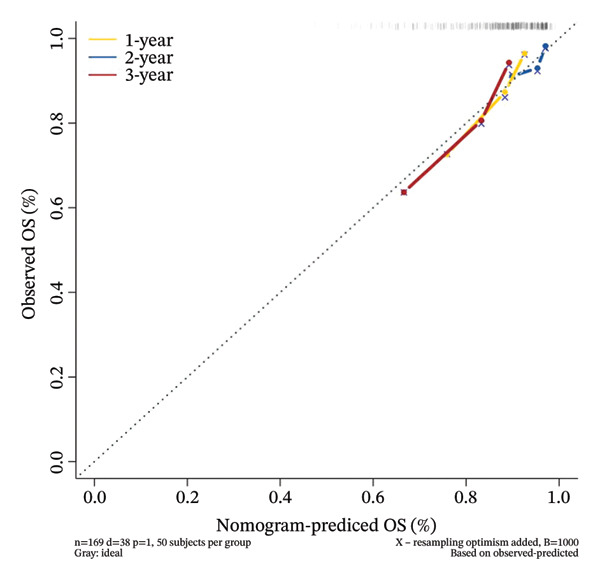
(e)

**Figure:**
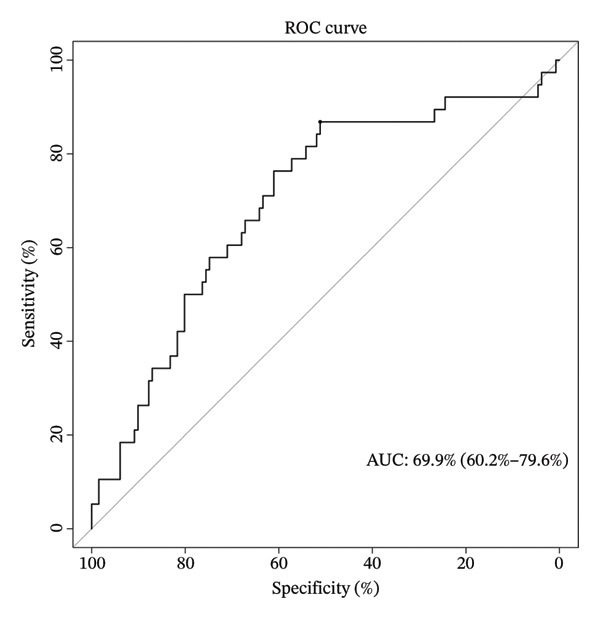
(f)

The nomogram’s potential as a clinically useful tool for personalized survival estimation in BC patients.

### 3.9. Prediction of Drug Response Based on Cuproptosis Risk Score

**FIGURE 8:**
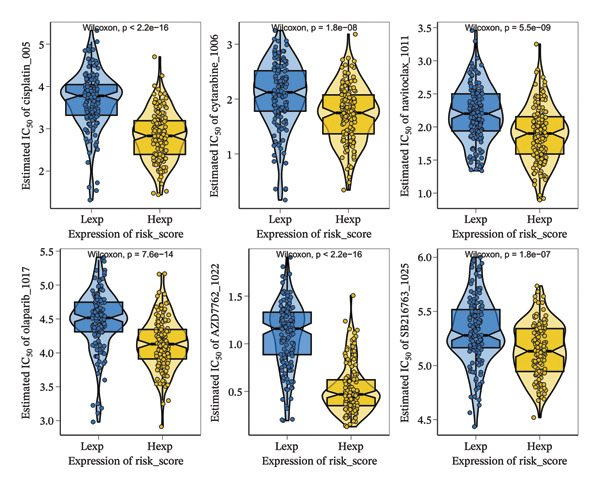
Drug sensitivity prediction based on the cuproptosis risk score. Violin plots show the estimated IC_50_ values of six chemotherapeutic agents—cisplatin, cytarabine, navitoclax, olaparib, AZD7762, and SB216763—in high‐risk (hexp) and low‐risk (lexp) groups. All six drugs exhibited significantly lower IC_50_ values in the high‐risk group (Wilcoxon test: *p* < 2.2 × 10^−16^ to *p* = 1.8 × 10^−7^), indicating enhanced sensitivity. These results support the potential of the risk score in predicting treatment response and informing precision chemotherapy.

Using the oncoPredict algorithm, we estimated the chemosensitivity of BC samples based on transcriptomic profiles in the GSE25055 training set. Significant differences in drug sensitivity were observed between the high‐risk and low‐risk groups, as stratified by the cuproptosis‐based risk score (Figure 8). Specifically, the high‐risk group showed significantly lower estimated IC_50_ values for six chemotherapeutic agents: Cisplatin (*p* < 2.2 × 10^−16^), cytarabine (*p* = 1.8 × 10^−8^), navitoclax (*p* = 5.5 × 10^−9^), olaparib (*p* = 7.6 × 10^−14^), AZD7762 (*p* < 2.2 × 10^−16^), and SB216763 (*p* = 1.8 × 10^−7^).

Patients in the high‐risk group may be more responsive to DNA‐damaging agents and apoptosis regulators, highlighting the model’s potential in guiding personalized therapy.

### 3.10. Expression Validation of Model Genes via RT‐qPCR

To functionally validate the key genes identified from the risk score model, we assessed the expression levels of CIRBP, INPP4B, IL6ST, and CCL20 across four human mammary cell lines (MCF10A, MCF‐7, BT‐474, and MDA‐MB‐231) using RT‐qPCR (Figure 9). These four genes were selected as representative model genes for experimental validation because they showed relatively strong statistical weight in the risk score model and have been previously reported to be biologically relevant to BC, including roles in tumor progression, immune regulation, and chemotherapy response [19–22]. RT‐qPCR results revealed cell line–specific expression patterns. For instance, INPP4B and IL6ST were highly expressed in MCF‐7, whereas CIRBP showed relatively high expression in BT‐474. In contrast, CCL20 was markedly upregulated in MDA‐MB‐231 compared to MCF10A.

FIGURE 9RT‐qPCR validation of model‐associated gene expression in breast cancer and normal mammary epithelial cell lines. Relative expression levels of CIRBP, INPP4B, IL6ST, and CCL20 were measured in MCF10A (nontumorigenic normal‐like mammary epithelial cells), MCF‐7 (luminal A–like breast cancer cells), BT‐474 (HER2‐positive breast cancer cells), and MDA‐MB‐231 (triple‐negative breast cancer cells). The expression patterns reflect subtype‐associated differences among breast cancer cell models.

**Figure:**
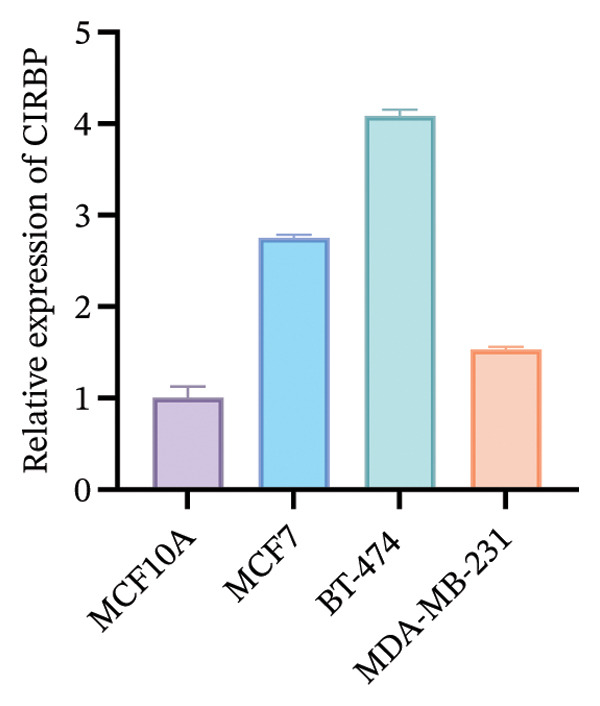
(a)

**Figure:**
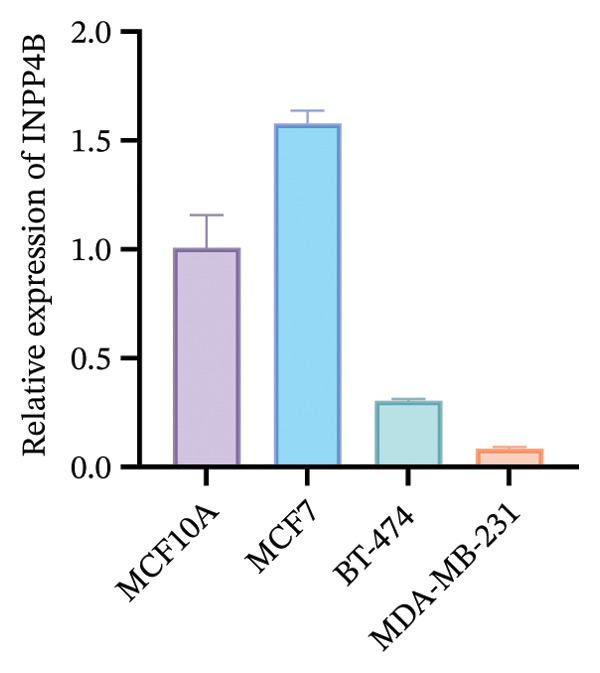
(b)

**Figure:**
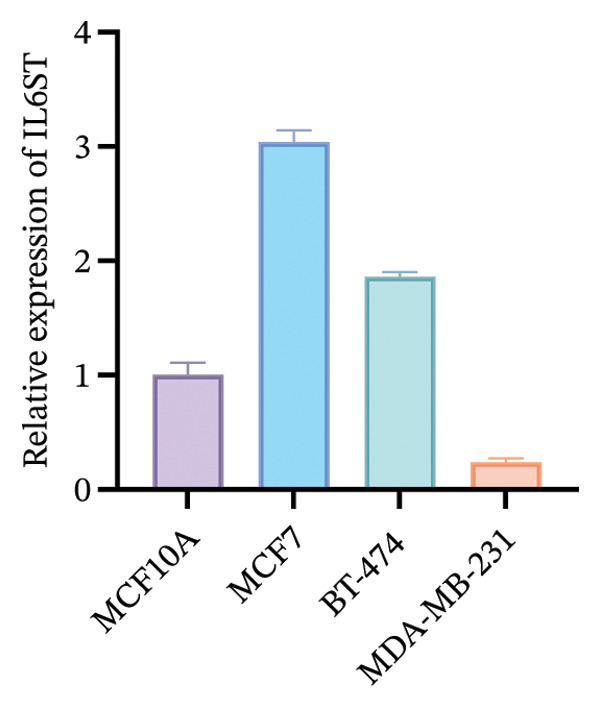
(c)

**Figure:**
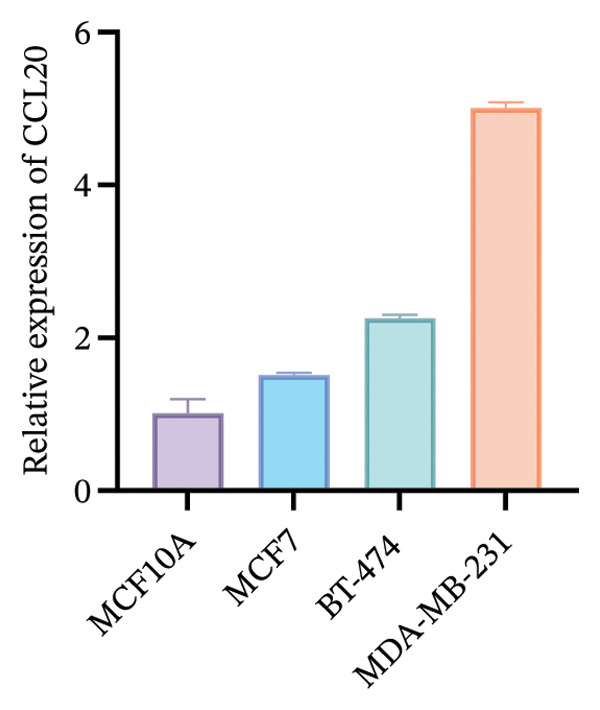
(d)

The expression of these model‐related genes may reflect molecular subtype differences and potentially contribute to the heterogeneity of chemoresistance and immune responses in BC.

## 4. Discussion

This study aimed to develop and validate a cuproptosis‐related gene signature for predicting the response of BC patients to NAC. By integrating transcriptomic data from TCGA and GEO, we identified 71 cuproptosis‐related genes and demonstrated the predictive power of the model in relation to pCR and RD. These findings suggest that cuproptosis‐related pathways may influence chemotherapy response through coordinated effects on mitochondrial metabolism, redox homeostasis, and tumor–immune interactions, thereby providing a mechanistic basis for the observed association between the risk score and NAC sensitivity in BC.

Using both GSEA and GSVA, our enrichment analyses consistently indicated that cuproptosis‐related genes were involved in immune‐related pathways, including monocyte chemotaxis and chemokine‐mediated signaling. GSEA evaluates pathway enrichment at the group level, whereas GSVA estimates pathway activity at the individual‐sample level. Therefore, concordant findings across both methods support the robustness of these enrichment results, while minor differences may reflect methodological sensitivity. These pathways are involved in immune cell recruitment and activation, which are critical to TME and play a major role in determining the efficacy of chemotherapy [23]. Among the 10 genes included in the model, CIRBP, INPP4B, IL6ST, and CCL20 emerged as key contributors. Their expression patterns were associated with chemotherapy response, and their functional roles in tumor progression, immune modulation, and drug resistance are supported by existing literature [24]. Kaplan–Meier survival analysis and ROC curves showed the model’s strong prognostic performance in both datasets. Notably, the model effectively stratified patients by their risk of recurrence, highlighting its potential utility in guiding NAC decision‐making. Recent studies have emphasized the need for more predictive biomarkers to guide NAC treatment, as the heterogeneity in treatment response remains a major clinical challenge [25, 26].

The ability to predict NAC response based on cuproptosis‐related genes offers a path toward personalized medicine in BC [27]. The model may help refine risk stratification for NAC response, particularly when interpreted in conjunction with established molecular subtypes and immune‐related features. As NAC is increasingly used for locally advanced and high‐risk BC, the need for reliable predictive models is crucial. PAM50 classification [28], which stratifies BC into molecular subtypes, has been shown to correlate with chemotherapy efficacy. In our study, the risk score was significantly associated with PAM50 subtypes, implying that our model integrates well with established clinical markers and may enhance their predictive value. Compared to PAM50 classification, which primarily reflects intrinsic molecular subtypes but does not explicitly capture metabolic stress responses or immune‐related heterogeneity, the cuproptosis‐based risk score provides a quantitative and continuous transcriptomic measure integrating metabolic and immune features associated with chemotherapy sensitivity, thereby offering complementary information when used alongside established subtype‐based classifiers in clinical practice. Notably, within the same cohorts analyzed, the cuproptosis‐based risk score showed consistent associations with pathologic response and survival outcomes that were not fully explained by PAM50 subtype distribution, supporting its potential additive value when used alongside established molecular classifiers. Rather than replacing established subtype‐based predictors, our model may serve as a complementary tool by integrating cuproptosis‐related transcriptional features with tumor immune context, thereby offering additional biological insight into NAC sensitivity beyond conventional classification systems. Immune infiltration analysis further revealed that the risk score was significantly correlated with the abundance of activated DCs and other immune cell subtypes, suggesting that cuproptosis‐related gene expression might influence immune composition within the TME. It should be noted that immune cell infiltration was inferred using the CIBERSORT algorithm based on bulk transcriptomic data, which estimates relative immune cell proportions rather than absolute counts. Therefore, these results should be interpreted as indicative associations rather than definitive measurements of immune cell composition. No additional immune deconvolution methods or publicly available single‐cell datasets were applied for orthogonal validation in the present study, and therefore, the observed associations should be interpreted with caution. This observation aligns with emerging evidence that tumor–immune interactions shape the chemotherapy response and offers a potential explanation for the model’s predictive power [29].

While this integrative approach provides valuable insights, the model, given its moderate predictive performance, is not intended to function as a standalone clinical decision‐making tool but rather as an adjunctive risk stratification framework to support NAC response assessment. First, the model requires validation in larger, prospective multicenter cohorts to ensure its applicability in routine clinical practice. Second, the predictive relevance of cuproptosis may vary across BC molecular subtypes, such as HER2‐positive disease and TNBC, warranting subtype‐specific investigations. Furthermore, although transcriptomic data provide important biological insights, the integration of additional omics layers including proteomics, epigenomics, and metabolomics may further enhance the robustness of the model by more comprehensively reflecting tumor complexity. Lastly, while the drug sensitivity analysis suggested potential therapeutic agents such as cisplatin and olaparib, these findings were derived from transcriptomic‐based in silico predictions and should be regarded as hypothesis‐generating, as transcriptome‐based IC_50_ estimates do not fully capture TME influences or pharmacokinetic factors that may affect treatment response in vivo.

## 5. Conclusion

Our study established and confirmed a cuproptosis‐based risk score model that robustly predicts NAC response in BC. The model integrates transcriptomic features with immune microenvironment characteristics and demonstrates consistent performance across independent datasets. Our findings support the utility of cuproptosis‐related genes as predictive biomarkers and underscore the potential of this model to guide personalized treatment strategies, ultimately improving clinical decision‐making in the neoadjuvant setting.

## Author Contributions

Lihai Zhang conceptualized and supervised the study, secured funding, and critically revised the manuscript. Jiao Wang contributed to data curation, formal analysis, and drafted the initial version of the manuscript. Baihong Tan participated in methodology design and data visualization. Xingquan Wang assisted with statistical analysis and bioinformatics interpretation. Zhenglong Luan contributed to experimental validation and bioinformatics interpretation.

## Funding

This study is the supported by Heilongjiang Provincial Health Commission Science and Technology Plan (20240404010074).

## Disclosure

All authors read and approved the final version of the manuscript.

## Ethics Statement

The authors have nothing to report.

## Consent

The authors have nothing to report.

## Conflicts of Interest

The authors declare no conflicts of interest.

## Supporting Information

Additional supporting information can be found online in the Supporting Information section.

## Supporting information


**Supporting Information** Supporting Figure 1. Enrichment analysis of cuproptosis survival genes. (A) GO enrichment analysis showing notable immune‐related biological processes, including monocyte chemotaxis, chemokine‐mediated signaling pathway, and lymphocyte migration. (B) KEGG analysis revealed enrichment in chemokine and cytokine receptor interactions, suggesting the involvement of immune‐regulatory mechanisms in NAC response.

## Data Availability

The data used to support the findings of this study are available from the TCGA‐BRCA dataset and the GEO dataset (GSE25055, GSE25065, and GSE25066).
