# Supplementary material for: Development and Validation of a Cuproptosis‐Based Risk Score Model for Predicting Neoadjuvant Chemotherapy Response in Breast Cancer: A Transcriptomic Analysis
Source: Breast J. 2026 May 29;2026:6097777. doi: 10.1155/tbj/6097777 (PMC13239323; doi:10.1155/tbj/6097777)
Supplement: Supplementary file 1 — Supporting Information Supporting Figure 1. Enrichment analysis of cuproptosis survival genes. (A) GO enrichment analysis showing notable immune‐related biological processes, including monocyte chemotaxis, chemokine‐mediated signaling pathway, and lymphocyte migration. (B) KEGG analysis revealed enrichment in chemokine and cytokine receptor interactions, suggesting the involvement of immune‐regulatory mechanisms in NAC response. [file TBJ-2026-6097777-s001.zip › Supplementary Table 1.docx]

Supplementary Table 1. Primer sequences used for qRT-PCR

| Gene | Forward (5′→3′) | Reverse (5′→3′) |
| --- | --- | --- |
| CIRBP | TGTTGGAGGGCTGAGTTTTG | TAACTGTCTCTGTAGGACCC |
| INPP4B | TGTCAGGAGTGCTCTTTTAGC | CAATCAGTTTGTCCACCATAGC |
| IL6ST | GTCATCTTCTATACGCACAGG | TATGTGCGTATGTGCGTATCAG |
| CCL20 | ATACGCACTAATGTGCTGTACC | TGTTTTGGATTTGCGCACACAG |
| GAPDH | AAGATCATCAGCAATGCCTCC | AGGTTTTTCTAGACGGCAGG |
